# Untargeted metabolomics of pulmonary tuberculosis patient serum reveals potential prognostic markers of both latent infection and outcome

**DOI:** 10.3389/fpubh.2022.962510

**Published:** 2022-11-15

**Authors:** Xuezhi Wang, Zhuhua Wu, Jincheng Zeng, Yuchuan Zhao, Chenchen Zhang, Meiling Yu, Wei Wang, Xunxun Chen, Liang Chen, Jiawen Wang, Liuyue Xu, Jie Zhou, Qiuchan Tan, Wenjing Wei, Yanxia Li

**Affiliations:** ^1^Foshan Fourth People's Hospital, Foshan, China; ^2^Center for Tuberculosis Control of Guangdong Province, Guangzhou, China; ^3^Dongguan Key Laboratory of Medical Bioactive Molecular Development and Translational Research, Guangdong Provincial Key Laboratory of Medical Molecular Diagnostics, Guangdong Medical University, Dongguan, China; ^4^Dongguan Key Laboratory of Medical Bioactive Molecular Development and Translational Research, Guangzhou Health Science College, Guangzhou, China

**Keywords:** latent TB infections (LTBI), tuberculosis, biomarkers, metabolomics, GC-MS, LC-MS/MS

## Abstract

Currently, there are no particularly effective biomarkers to distinguish between latent tuberculosis infection (LTBI) and active pulmonary tuberculosis (PTB) and evaluate the outcome of TB treatment. In this study, we have characterized the changes in the serum metabolic profiles caused by *Mycobacterium tuberculosis* (Mtb) infection and standard anti-TB treatment with isoniazid–rifampin–pyrazinamide–ethambutol (HRZE) using GC-MS and LC-MS/MS. Seven metabolites, including 3-oxopalmitic acid, akeboside ste, sulfolithocholic acid, 2-decylfuran (4,8,8-trimethyldecahydro-1,4-methanoazulen-9-yl)methanol, d-(+)-camphor, and 2-methylaminoadenosine, were identified to have significantly higher levels in LTBI and untreated PTB patients (T0) than those in uninfected healthy controls (Un). Among them, akeboside Ste and sulfolithocholic acid were significantly decreased in PTB patients with 2-month HRZE (T2) and cured PTB patients with 2-month HRZE followed by 4-month isoniazid-rifampin (HR) (T6). Receiver operator characteristic curve analysis revealed that the combined diagnostic model showed excellent performance for distinguishing LT from T0 and Un. By analyzing the biochemical and disease-related pathways, we observed that the differential metabolites in the serum of LTBI or TB patients, compared to healthy controls, were mainly involved in glutathione metabolism, ascorbate and aldarate metabolism, and porphyrin and chlorophyll metabolism. The metabolites with significant differences between the T0 group and the T6 group were mainly enriched in niacin and nicotinamide metabolism. Our study provided more detailed experimental data for developing laboratory standards for evaluating LTBI and cured PTB.

## Introduction

Tuberculosis (TB) is one of the most common communicable diseases worldwide, until the coronavirus, TB was the main cause of death from a single infectious agent. According to the WHO Global TB Report 2021, about 1.5 million people died from TB in 2020. A quarter of the global population was latently infected with *Mycobacterium tuberculosis* (Mtb) but remains asymptomatic, 5–15% of those individuals are likely to develop active TB (1). Early diagnosis has been recognized as the pillar to achieve the end of the TB epidemic; thus, the development of accurate, rapid, and easy diagnostic tools to improve diagnosis is required urgently.

Currently, the diagnosis of TB cases mainly depends on medical history, physical examination, imageological examination, and other laboratory tests. The traditional methods demonstrate some limitations in determining whether patients with TB are cured, as well as in identifying TB from latent tuberculosis infection. About 85% of TB patients can be successfully treated with a standardized 6-month anti-TB treatment regimen (2-month intensive phase plus a 4-month continuation phase) (2). However, due to the lack of a rapid and accurate method to evaluate the anti-TB efficacy, ~14% of discharged patients are not completely cured (3). A major cause resulting from interrupted treatment is the development of drug-resistant TB, it also increases the risk of TB transmission and spread in a community (4). Besides the cured TB patients, no well-validated or specific biomarkers can differentiate effectively latent tuberculosis infection from active TB. The WHO recommends that an interferon (IFN)-γ release assay (IGRA) or a tuberculin skin test (TST) can be used to screen for TB infection (5). However, neither the IGRAs nor the TST can discriminate between latent tuberculosis infection and active TB (5–7). Consequently, a panel of rapidly measured biomarkers with high diagnostic accuracy is crucial for global TB control.

Metabolites are small molecules that represent ongoing biological processes and may provide insights into the mechanisms that underlie the disease process as well as disease progression (8). Due to their special characteristics, metabolites have become potential disease biomarkers for disease (9, 10). In recent years, metabonomics has been widely used in disease research because it provides a more precise method to detect changes in metabolism (11). In a recent study, a 4-differential metabolite in combination can be used as a potential biomarker to cure TB (3). In further a prospective multisite study across Subsaharan Africa, a trans-African metabolic biosignature for TB was found to predict the progression of TB at 69% sensitivity and 75% specificity on blinded test samples and in external data sets. Among the main analytical methods, mass spectrometry (MS) displays its high sensitivity and high throughput and has been widely used for identifying specific metabolites (13, 14). Currently, metabolomics technology is still not perfect, it is not yet possible to detect all compounds with one technology and different detection platforms are required. LC-MS/MS is commonly used for detailed analysis of natural compounds, such as serum, plasma, urine, and disease samples. GC-MS has an advantage over LC-MS/MS in the analysis of volatile and thermally stable metabolites. Therefore, LC-MS/MS combined with GC-MS could identify significantly altered metabolites as comprehensively as possible.

Here, we performed a well-powered, untargeted TB-associated serum metabolomics assessment by integrating the GC-MS and LC-MS/MS assays. We investigated the impact of Mtb infection on the serum metabolome and characterized the changes induced by front-line TB antibiotics on the composition of the serum metabolome at different time points, to provide a set of candidates for predicting the Mtb infection and potential outcome.

## Materials and methods

### Subjects in this study and sample collection

The serum samples used in this study were collected from the Major Infectious Disease Prevention and Control of the National Science and Technique Major Project and preserved by the Biobank of the Center for Tuberculosis Control of Guangdong Province. The volunteers in this study were enrolled and subjected to the analysis using the IFN-γ release assay (QuantiFERON-TB Gold In-Tube (QFT), Qiagen, CA, USA) along with clinical, microbiological, and radiographical examinations. The criteria for enrollment were as follows: (1) patients with active TB (ATB group in this study) showed clinical and radiographical features of tuberculosis and were confirmed by sputum smear or culture. Moreover, they did not receive anti-TB treatment before sample selection. (2) Standard anti-TB treatment includes RIF, INH, PZA, and EMB for 2 months, followed by RIF and INH for additional 4 months. After 6 month-treatment, PTB patients were considered cured if they meet the following conditions: chest X-ray or CT examination of TB symptoms disappeared, negative sputum test or sputum smear positive TB patients turned negative and symptoms improved. All active TB patients receive periodic follow-up appointments while on treatment. Exclusion criteria included a history of antibiotic or probiotic treatment more than 1 week within the previous 8 weeks. (3) All the controls (IGRA- and IGRA+) were with TB-resembling coughing symptoms and normal X-rays but were culture-negative and without other clinical TB symptoms and TB contact history. The clinical characteristics of the groups are given in Table 1 and Supplementary Table 1. All subjects with diabetes mellitus, HIV infection, hepatitis B, metabolic diseases, autoimmune diseases, and malignant tumors were excluded. Peripheral blood samples were obtained by venipuncture from all subjects and collected in an aseptic vacuum blood collection tube and stored at 2~8°C. The serum was centrifugated, collected, and stored in an −80°C refrigerator for cryopreservation. This study was performed in compliance with the Declaration of Helsinki. The study was approved by the Ethics Committee of the Center for Tuberculosis Control of Guangdong Province, China. Written informed consent was obtained from all subjects before blood sample collection.

**Table 1:** Characteristic of pulmonary TB patients during the therapy, latent tuberculosis infection and healthy controls.

	**Un**	**LT**	**T0**	**T2**	**T6**	**χ^2^**	***P*-value**
Age, years range (mean ± SD)	20–53 34.8 ± 10.35	18–57 38.0 ± 10.64	18–59 35.76 ± 12.61	18–59 35.76 ± 12.6	18–59 35.76 ± 12.6		0.710^a^
Total Subjects (male)	15 (8)	16 (11)	17 (13)	17 (13)	17 (13)	1.967	0.374^b^

Un, Healthy controls; T0, Untreated PTB; T2, PTB with anti-TB chemotherapy for 2 months; T6, Cured PTB with anti-TB chemotherapy for 6 months; LT, Latent tuberculosis infection.

Given that T0, T2, and T6 belonged to the same group of patients, the statistical analysis of age and sex differences among the multiple groups presented here was performed in Un, LT, and T0.

a One-way ANOVA test.

b Chi-square test.

### LC-MS/MS metabolite extraction

Extraction of serum metabolites for LC-MS/MS analysis was performed as previously described (15). Briefly, another 100 μL of the above serum samples were taken and mixed with 400 μL prechilled methanol by well vortexing. The samples were incubated on ice for 5 min and then were centrifuged at 15,000 rpm, 4°C for 5 min. Some of the supernatants were diluted to a final concentration containing 60% methanol by LC-MS grade water. The samples were subsequently transferred to a fresh Eppendorf tube with a 0.22 μm filter and then were centrifuged at 15,000 *g*, 4°C for 10 min. A total of 60 μL of each sample were pipetted and mixed to form a QC sample. Finally, the filtrate was injected into the LC-MS/MS system analysis.

### GC-MS metabolite extraction

Metabolite extraction for GC-MS analysis was performed according to previously published procedures with some modifications (16). A total of 100 μL serum sample was mixed with 300 μL prechilled methanol and 10 μL fluorophenylalanine, followed by vortexing and ultrasound concussion. The supernatant was carefully pipetted into a 1.5 mL EP tube (5). All the samples were dried completely in the vacuum concentrator without heating. A total of 60 μL Methoxyamination hydrochloride (20 mg/mL in pyridine) was added and incubated for 30 min at 80°C. A total of 80 μL of the BSTFA [N,O-Bis(trimethylsilyl) trifluoroacetamide] regent (1% TMCS, v/v) (Trimethylchlorosilane) was added and incubated at 70°C for 1.5 h. All the samples were analyzed by a gas chromatography system coupled with a Pegasus HT time-of-flight mass spectrometer (GC-MS). A total of 60 μL of each sample were pipetted and mixed to form a QC sample.

### LC-MS/MS analysis

LC-MS/MS analysis was operated in both positive and negative ion modes with the parameters optimized according to previously published procedures with some modifications (17). LC-MS/MS analyses were performed using a Vanquish UHPLC system (Thermo Fisher) coupled with an Orbitrap Q Exactive series mass spectrometer (Thermo Fisher). Samples were injected into the Hyperil Gold column (100 × 2.1 mm, 1.9 μm) using a 16-min linear gradient at a flow rate of 0.2 mL/min. The eluents for the positive polarity mode were eluent A (0.1% FA in Water) and eluent B (Methanol). The eluents for the negative polarity mode were eluent A (5 mM ammonium acetate, pH 9.0) and eluent B (Methanol). The solvent gradient was set as follows: 1.5 min, 2% B; 12.0 min, 100% B; 14.0 min, 100% B; 14.1 min, 2% B; 16 min, 2% B. The flow rate was 0.2 mL/min. Q Exactive mass series spectrometer was operated in positive/negative polarity mode with a spray voltage of 3.2 kV, a capillary temperature of 320°C, a sheath gas flow rate of 35 arb, and an aux gas flow rate of 10 arb.

### GC-MS analysis

Agilent 7,890 gas chromatograph system coupled with a Pegasus HT time-of-flight mass spectrometer was used for GC-MS analysis (16). The system utilized a DB-5MS capillary column coated with 5% diphenyl cross-linked with 95% dimethylpolysiloxane (30 m × 250 μm inner diameter, 0.25 μm film thickness; J&W Scientific, Folsom, CA, USA). A 1 μL aliquot of the analyte was injected in splitless mode. Helium was used as the carrier gas, the front inlet purge flow was 3 mL/min, and the gas flow rate through the column was 1 mL/min. The initial temperature was kept at 50°C for 1 min, then raised to 310°C at a rate of 20°C min^−1^, then kept for 6 min at 310°C. The injection, transfer line, and ion source temperatures were 280, 280, and 250°C, respectively. The energy was −70eV in electron impact mode. The mass spectrometry data were acquired in full-scan mode with the *m*/*z* range of 50–500 at a rate of 12.5 spectra per second after a solvent delay of 4.78 min.

### Data processing and analysis

The raw data files of LC-MS/MS and GC-MS were processed by the software Compound Discoverer 3.1 (CD) (18) and Chroma TOF (V4.3X, LECO) (19), respectively. Initially, the data were performed peak alignment after filtrating by retention time and mass-to-charge ratio. Next, the exact molecular mass of the compounds utilized was determined by the mass-to-charge ratio in the high-resolution XIC charts. Meanwhile, the molecular formulas were predicted based on the mass deviation and adduct ion information. The metabolites in the biological system were identified by matching the fragment ion, collision energy, as well as other information of each compound, respectively, in the mzCloud database and LECO-Fiehn Rtx5 database. Subsequently, the compounds in the QC sample with a value of Coefficient of Variance (CV) < 30% were applied as the final identification results for subsequent analysis. The Pearson correlation coefficient between QC samples was calculated based on the peak area value, the higher the correlation of QC samples, the better the stability of the whole detection process and the higher the data quality. The multivariate statistical methods, such as PCA and Partial Least Squares Discrimination Analysis (PLS-DA), were used to perform dimensionality reduction and regression analysis on the multi-dimensional data based on preserving the original information to the greatest extent. To annotate the function and classification of the identified metabolites, the databases KEGG (http://www.genome.jp/kegg/), HMDB (http://www.hmdb.ca/), and LIPIDMAPS (http://www.lipidmaps.org/) (20) were used in this study. Student's *t*-test was performed for parametric data between two groups. The statistical difference among multiple groups was analyzed using analysis of variance (ANOVA) for parametric data and the Kruskal-Wallis test for non-parametric data. A Chi-square test was performed for the composition ratios. The receiver operating characteristic curve (ROC) was drawn using SPSS (version 26.0, USA) software. *P* ≤ 0.05 was considered significant.

## Results

In this research, we enrolled 17 pulmonary TB patients without HRZE treatment (T0 group) and 31 healthy controls, including 15 volunteers without Mtb (Un group) and 16 volunteers with latent Mtb infection (LT group), to study the relationship between tuberculosis and serum metabolites. Furthermore, to detect the effect of periodic HRZE treatment, we tracked the serum metabolome changes in these TB patients with standard anti-TB therapy for 2 months (T2) and 6 months (T6), respectively. Detailed characteristics of recruited participants are shown in Table 1 and Supplementary Table 1.

### Raw data pre-processing

We adopted an untargeted metabolomic approach using GC-MS and LC-MS/MS to cohorts of our serum samples. To study the variation of metabolites among different groups, we filtered out annotated peaks in the alignment table that presented in < 50 % of the samples in each group. After this filtering, a total of 2,563 annotated peaks were commonly detected by all three platforms, while 1,456 annotated peaks by LC-MS/MS (+), 862 annotated peaks by LC-MS/MS (-), and 245 annotated peaks by GC-MS. These peaks were aligned using KEGG, HMDB, and LIPID MAPS databases to annotate the functional characteristics and classification of different metabolites. Data containing respective RT, relative log intensities, the variable importance in projection (VIP), and *P*-value was used for subsequent group comparisons.

### Active TB can affect human serum metabolome

**Figure 1:**
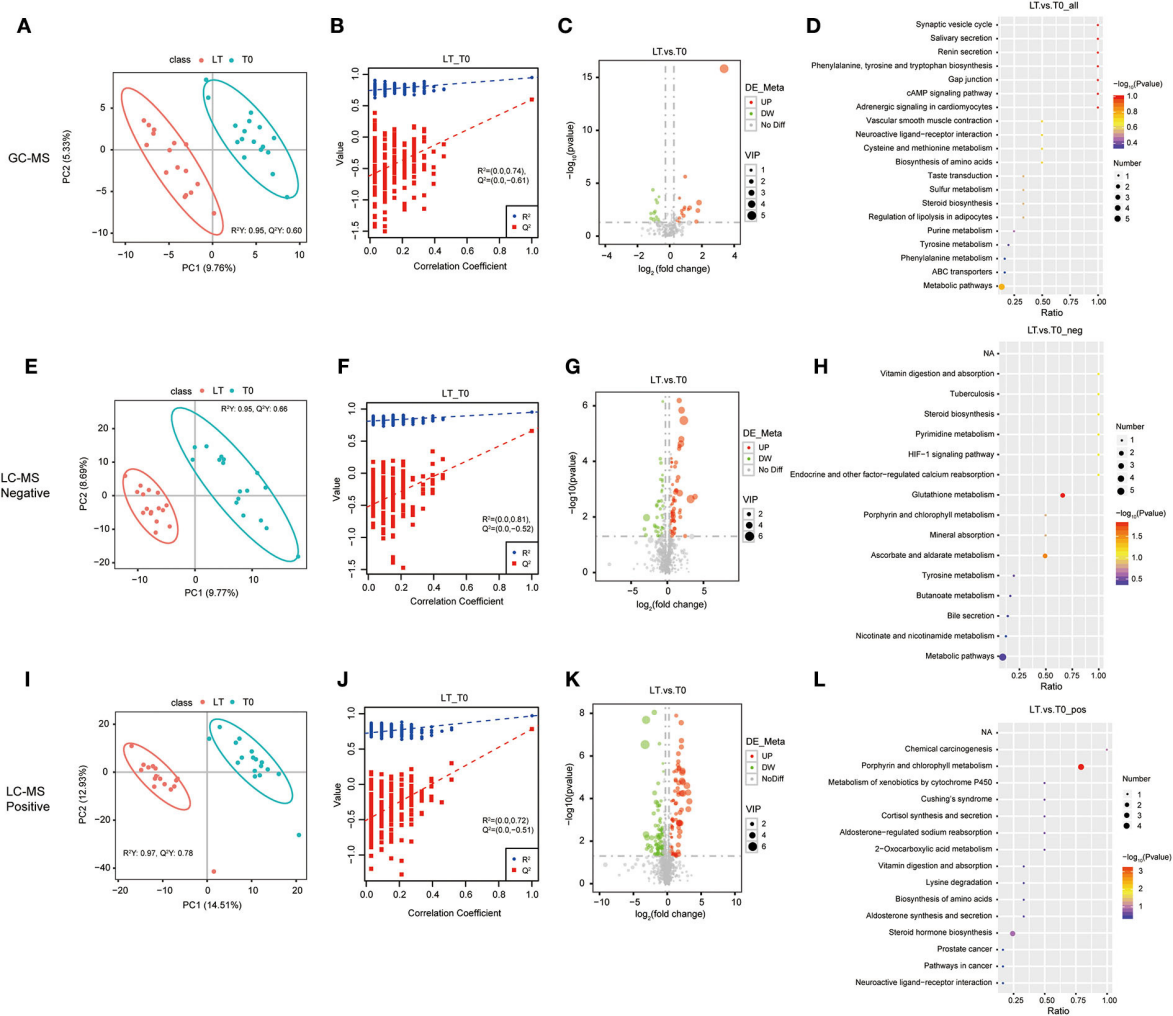
Identification of serum metabolites between latent tuberculosis infection (LT) and the untreated PTB group (Un). The PLS-DA model for the LT/Un group in GC-MS **(A)**, LC-MS/MS (–) **(E)**, and LC-MS/MS (+) **(I)**. The permutation test results for the LT/Un group in GC-MS **(B)**, LC-MS/MS (–) **(F)**, and LC-MS/MS (+) **(J)**. Volcanic map of differential metabolites for the LT/Un group in GC-MS **(C)**, LC-MS/MS (–) **(G)**, and LC-MS/MS (+) **(K)**. The abscissa: the fold change of LT/Un group (base 2 logarithm). The ordinate: the *P*-value of LT/Un group (base 10 logarithm). Red: significantly upregulated metabolites. Green: significantly downregulated metabolites. Gray: non-significant differential metabolites. Pathway analysis of differential metabolites for the LT/Un group in GC-MS **(D)**, LC-MS/MS (**-**) **(H)**, and LC-MS/MS (+) **(L)**.

To assess the effect of Mtb infection on serum metabolome, the metabolites in serum samples were analyzed among the Un, LT, and T0 groups. To separate the important features of significant differences between these three groups, the PLS-DA model was used to eliminate the overfitting of test models and evaluate the statistical significance of the models. The PLS-DA score plots showed that the characteristics of metabolites were able to clearly distinguish the T0 group from the LT group (Figures 1A,E,I) and the Un group (Supplementary Figure 1). The results of the permutation test of the PLS-DA model showed that the LT group and Un group (Figures 1B,F,J) demonstrated a robust prediction performance and no over-fitting phenomenon, as well as in the LT group and the T0 group (Supplementary Figure 1).

To further determine which metabolites were significantly affected by active Mtb, we integrated the LC-MS/MS and GC-MS metabolomics data to investigate significant features among the Un group, LT group, and T0 group. Differential metabolites were defined as those that showed a fold change >2.0 or < 0.50 in relative abundance and a *p*-value < 0.05. Based on these criteria, there were 160 metabolites with different abundance between the LT group and the T0 group (Supplementary Table 2), and 254 metabolites between the T0 group and Un group (Supplementary Table 3), respectively. In comparison between the LT group the and T0 group, 77 metabolites were upregulated (>2-fold) and 83 metabolites were downregulated (< 0.50-fold) in the LT group. There were 124 upregulated metabolites (>2-fold) and 128 downregulated metabolites (< 0.5-fold) in the T0 group when compared with the Un group. The differential metabolites between the LT group and the T0 group (Figures 1C,G,J), the Un group, and the T0 group (Supplementary Figure 1) were further illustrated in a volcano plot. Finally, we performed the pathway enrichment analysis for the selected metabolites. Metabolic pathway analysis [GC-MS, LC-MS/MS (+) and LC-MS/MS (–)] shows that differential metabolites are grouped in glutathione metabolism (*p*-value = 0.0145), ascorbate and aldarate metabolism (*p*-value =0.0279), and porphyrin and chlorophyll metabolism (*p*-value = 0.0004) between LT group and T0 group (Figures 1D,H,K,L and Supplementary Table 8). Besides, porphyrin and chlorophyll metabolism, bile secretion (*p*-value = 0.0006), primary bile acid biosynthesis (*p*-value = 0.0307), and mineral absorption (*p*-value = 0.0453), cholesterol metabolism (*p*-value =0.0453) were also significantly enriched between Un group and T0 group (Supplementary Figure 2 and Supplementary Table 8). These results further confirm that differential metabolites in serum have the potential to predict Mtb infection.

### Anti-TB therapy results in some significant changes in serum metabolome

**Figure 2:**
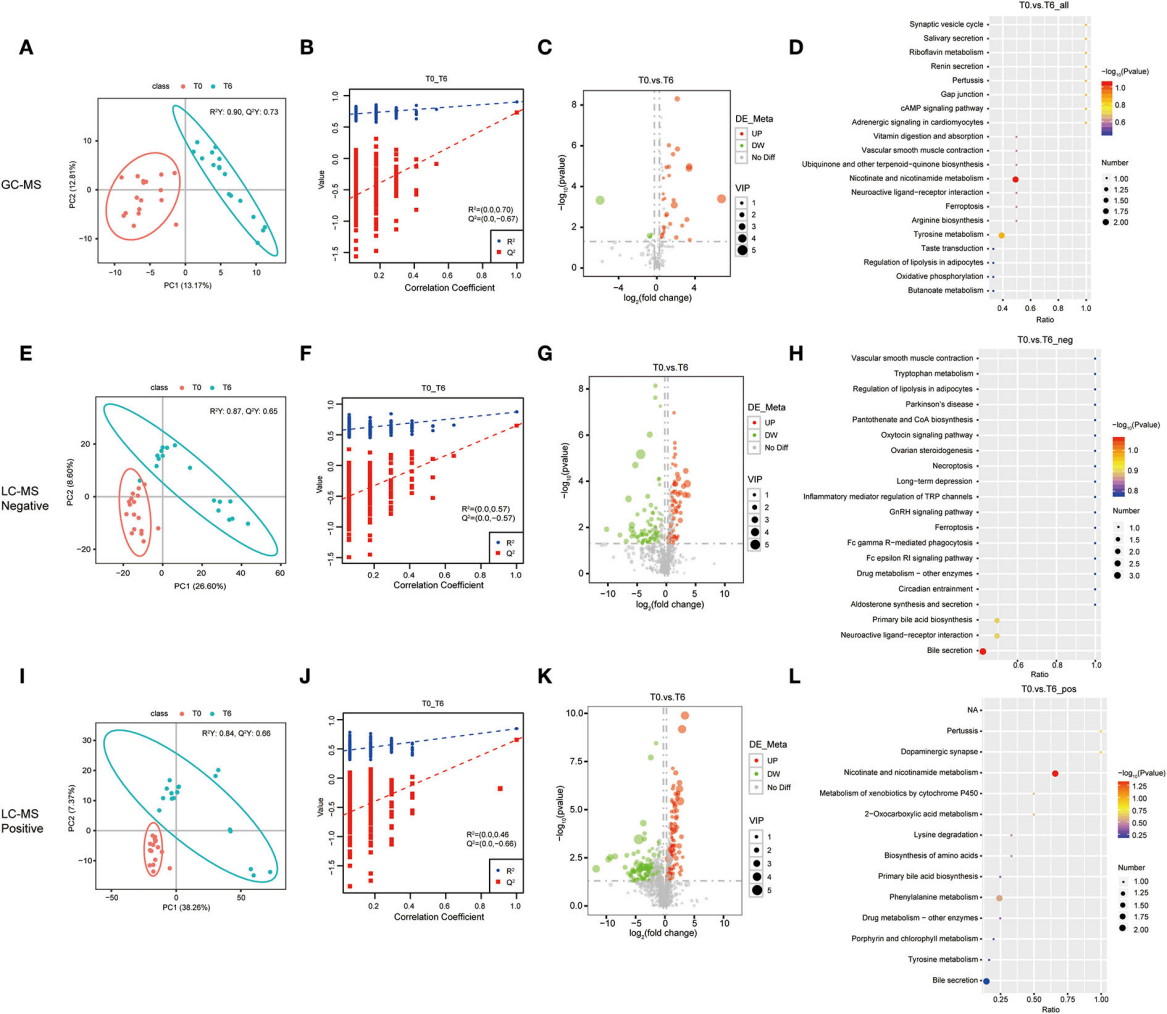
Identification of serum metabolites between the cured PTB group (T6) and the untreated PTB group (T0). The PLS-DA model for the T0/T6 group in GC-MS **(A)**, LC-MS/MS (–) **(E)**, and LC-MS/MS (+) **(I)**. The permutation test results for theT0/T6 group in GC-MS **(B)**, LC-MS/MS (–) **(F)**, and LC-MS/MS (+) **(J)**. Volcanic map of differential metabolites for the LT/Un group in GC-MS **(C)**, LC-MS/MS (–) **(G)**, and LC-MS/MS (+) **(K)**. The abscissa: the fold change of LT/Un group (base 2 logarithm). The ordinate: *P*-value of T0/T6 group (base 10 logarithm). Red: significantly upregulated metabolites. Green: significantly downregulated metabolites. Gray: non-significant differential metabolites. Pathway analysis of differential metabolites for the T0/T6 group in GC-MS **(D)**, LC-MS/MS (–) **(H)**, and LC-MS/MS (+) **(L)**.

To explore the relevance of serum metabolome with HRZE treatment, and the temporal evolution of the selected predictors, longitudinal data were acquired from 17 TB patients with standard therapy. Similarly, the PLS-DA score map (Figure 2) revealed that the T6 group can be demarcated from the T0 group. The permutation test showed that the performance of PLS-DA model data of the T0 group and the T6 group was consistent with that of the standard parameters (Figure 2), as well as the T0 group and the T2 group (Supplementary Figure 2). Therefore, it can be effectively and reliably applied to detect differences in metabolic profiles related to the potential outcome. Student's *t*-test was used to screen differential metabolites between T0 groups and T6 groups. Comparing the T0 group to the T6 group, we found that 18 features were screened by GC-MS, 216 features by LC-MS/MS (+), and 127 features by LC-MS/MS (–) (Supplementary Table 4), including 150 upregulated and 211 downregulated compounds. Selected compounds were displayed in the form of volcano plots (Figures 2C,G,K). Between the T0 groups and T2 groups, 13 features were screened by GC-MS, 70 features by LC-MS/MS (+), and 63 features by LC-MS/MS (–) (Supplementary Table 5), among which, 76 were upregulated and 70 were downregulated. Metabolic pathways enrichment is shown in Figure 2 between the T0 group and the T6 group, differential metabolites were mainly enriched in nicotinate and nicotinamide metabolism and bile secretion pathway. Besides, some amino acids, such as tyrosine metabolism, and phenylalanine metabolism were also significantly enriched.

### Screening of differential metabolites as potential biomarkers

**Figure 3:**
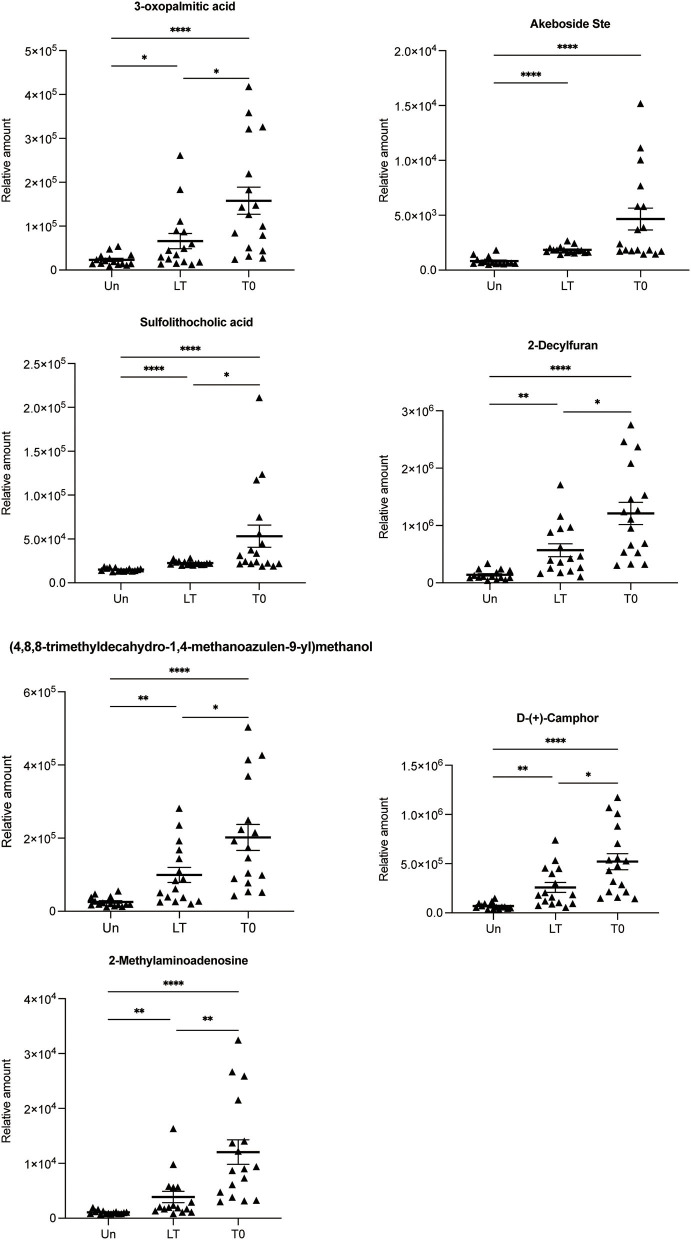
Relative abundance of seven differential metabolites 3-oxopalmitic acid, akeboside ste, sulfolithocholic acid, 2-decylfuran, (4,8,8-trimethyldecahydro-1,4-methanoazulen-9-yl) methanol, d-(+)-camphor, and 2-methylaminoadenosine. The relative abundance of each metabolite in the serum from the untreated PTB (T0) and latent tuberculosis infection (LT) was significantly higher than that of the healthy control (Un) using the Kruskal–Wallis test and corrected for multiple comparisons by controlling the False Discovery Rate. *q < 0.05; **q < 0.01; ****q < 0.0001.

Next, the abnormally abundant metabolites in the T0 group compared with the other two groups were sought through screening all differential metabolites (FC > 2.0, VIP > 1.0, and *P* < 0.05) in the Un group, the LT group, and the T0 group. Comparison of T0 with the other two groups (LT vs. T0 and Un vs. T0) showed that the relative amount of 72 overlapping metabolites had changed dramatically in the serum of T0 patients. Table 2 shows the top 20 differential metabolites between the three groups, and these are listed in order of fold change and significance level. The total list of metabolites can be found in Supplementary Table 6. Furthermore, we screened seven metabolites that presented the same trends when compared with three groups, including sulfolithocholic acid, 2-decylfuran, (4,8,8-trimethyldecahydro-1,4-methanoazulen-9-yl)methanol, D-(+)-camphor, 2-methylaminoadenosine, 3-oxopalmitic acid, and akeboside Ste (Figure 3).

**Table 2:** Top 20 differential serum metabolites of T0 patients identified by LC-MS/MS and GC-MS, compared to LT and Un individuals.

**No**	**Name_des**	**Un/T0**			**LT/T0**			**Trend**
		**Fold change**	***p*-value**	**VIP**	**Fold change**	***P*-value**	**VIP**	
1	2-Methylaminoadenosine	0.08869288	7.6354E-10	4.01165828	0.32183336	0.00014766	3.15312305	↑
2	Carmustine	0.08972882	6.1405E-07	5.36149848	0.10362071	2.9201E-07	6.7564025	↑
3	2-Mercaptobenzothiazole	0.09599409	4.2994E-08	4.79467767	0.1142865	2.0319E-08	5.98713527	↑
4	2-Decylfuran	0.11522464	7.6919E-10	3.85942897	0.47028435	0.00494403	2.08491179	↑
5	(4,8,8-trimethyldecahydro-1,4-methanoazulen-9-yl)methanol	0.12689437	5.1544E-09	3.50374746	0.49206327	0.01133179	2.00191124	↑
6	D-(+)-Camphor	0.13339036	8.9712E-10	3.47389398	0.49728562	0.00673748	1.95060669	↑
7	3-oxopalmitic acid	0.14779064	8.9333E-07	3.10115364	0.41789084	0.00686639	2.40303662	↑
8	Pivagabine	0.15975548	0.03742746	1.41310153	0.13634797	0.0153759	2.26464813	↑
9	Thiolutin	5.76453916	1.1133E-06	4.72982861	4.86432478	3.3855E-06	6.19438115	↓
10	Akeboside Ste	0.17761794	5.0832E-07	2.72736425	0.39555739	0.00583788	1.56591184	↑
11	Benzquinamide	5.43411528	0.00023544	2.28109023	9.63323124	0.00013558	4.18646907	↓
12	(9cis)-Retinal	0.19175918	0.00014079	2.39998295	0.3651436	0.04168866	1.81045086	↑
13	Vitamin C	4.85885066	2.1287E-05	3.37433699	3.66201592	2.3383E-05	4.36822211	↓
14	2,3,-dihydroxybenzoylserine	0.20816884	2.7158E-06	2.63686566	0.27423189	5.4365E-05	3.04325781	↑
15	Dcebio	4.72077327	4.1241E-07	3.46112538	3.916837	1.4456E-06	4.4541779	↓
16	Dexamethasone tebutate	4.61437664	1.4492E-11	2.72745654	4.24282632	3.7766E-07	3.25382949	↓
17	Maleic acid	4.37259767	6.7671E-05	2.8486705	3.83535948	1.5916E-05	3.99476391	↓
18	Methyprylon	0.23096774	0.03419834	1.21199933	0.21277934	0.02037897	1.79394108	↑
19	Seratrodast	4.1954909	0.00421687	1.79337805	8.50637327	5.1876E-05	4.29763145	↓
20	Methional	0.2479882	1.7002E-08	2.68873807	0.26589941	8.9786E-09	3.51678769	↑

**Figure 4:**
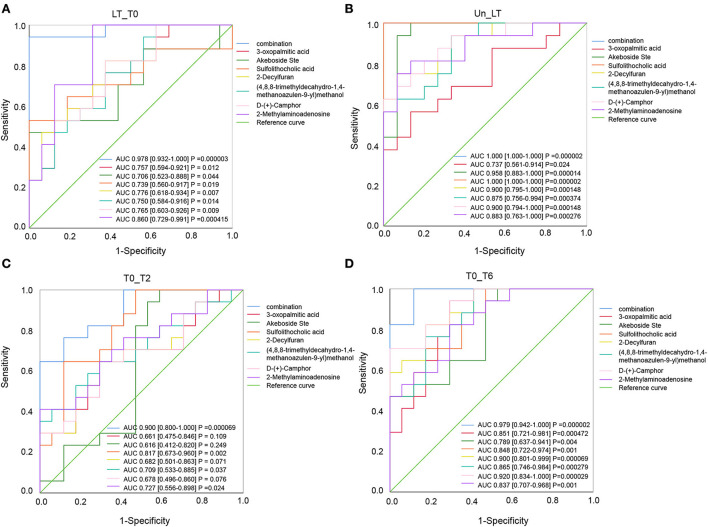
ROC curves are used to identify differential metabolites. The curve analysis of the seven metabolites shows that the combined diagnostic model performed well in distinguishing LT from T0 (AUC = 0.978) **(A)** and Un (AUC = 1.000) **(B)**. Compared with group T2 **(C)**, the combined diagnostic model could better distinguish T0 from T6 (AUC = 0.979) **(D)**.

The discriminative ability of these selected metabolites was evaluated by ROC curves. The seven differential metabolites mentioned above were used as input variables for multiple logistic regression analysis. The ROC curves of seven differential metabolites for distinguishing LT from T0 or Un are shown in Figure 4. When distinguishing the LT group from the T0 group, except that 2-methylaminoadenosine exhibited excellent efficiency with AUC values of 0.860 (95% CI 0.729–0.991), the remaining six differential metabolites had limited efficacy (AUC < 0.8) as a biomarker. When distinguishing the LT group from the Un group, 2-decylfuran, d-(+)-camphor, akeboside ste, and sulfolithocholic acid showed excellent performance with AUC of 0.900 (95% CI 0.795–1.000), AUC of 0.900 (95% CI 0.794–1.000), AUC of 0.958 (95% CI 0.883–1.000), and AUC of 1 (95% CI 1.000–1.000), respectively. Then, we further used multiple logistic regression to analyze the efficacy of the seven metabolite combination. ROC analysis showed that results were very good, indicating that these seven combinations could be used to represent the most suitable biomarker group for the differentiation of PTB patients from the healthy controls.

In the same way, we are looking forward to screening differentially abundant metabolites as potential biomarkers for the cured PTB group and the untreated PTB group. We sought the metabolites which expressed abnormally in T0 but recovered after anti-tuberculosis treatment by investigating all differential metabolites among the Un/T0 group, the T0/T2 group, and the T0/T6 group. A total of 18 overlapping metabolites were significantly differentially expressed in the serum of T0 patients (Table 3). In addition, we also noticed that after treatment, especially 6-month anti-TB therapy, the seven serum metabolites expressed abnormally in T0 (Figure 3) significantly decreased and were close to the level of the healthy group, further demonstrating the potential of these metabolites as TB-related biomarkers (Supplementary Figure 3).

**Table 3:** Metabolites with significant changes in the relative amount identified by LC-MS/MS and GC-MS among the Un group, T0 group, T2 group, and T6 group.

**No**.	**Differential Metabolites**	**Un/T0**			**T0/T2**			**T0/T6**		
		**FC**	***p*-value**	**VIP**	**FC**	***p*-value**	**VIP**	**FC**	***p*-value**	**VIP**
1	(2R)-2-Hydroxy-3-(phosphonooxy)propyl (11Z)-11-docosenoate	2.23074	4.00E-11	1.48559	0.48008	0.01504	1.00738	0.26091	0.00981	1.62940
2	Akeboside ste	0.17762	5.08E-07	2.72736	2.23626	0.020102	1.27419	2.88922	0.00117	1.22118
3	Alpha-ketoglutaric acid	0.00919	0.0004	5.63162	80.47541	0.00060	6.11196	108.83	0.00040	5.01282
4	Benzylamine	0.41113	2.88E-05	1.54977	2.19781	7.67E-05	1.63210	2.4323	2.88E-05	1.44356
5	Cellobiose	0.09573	1.03E-05	3.59862	10.44709	1.03E-05	4.04631	10.44666	1.03E-05	3.32044
6	Dehydrocholic acid	0.35737	0.01569	1.580281	3.09395	0.002455	2.65723	3.78539	0.00337	2.26530
7	Guanidinosuccinic acid	0.22328	1.43E-06	2.65049	2.72994	7.46E-05	2.39798	4.47863	1.43E-06	2.47196
8	Levoglucosan	0.16025	0.00827	1.94374	6.24032	0.00827	2.14569	6.2403	0.00827	1.71159
9	Lyxonic acid, 1,4-lactone	0.31000	2.58E-06	2.06159	3.22580	2.58E-06	2.36436	3.2258	2.58E-06	1.90368
10	Medroxyprogesterone 17-acetate	2.32418	0.00807	1.49834	8.76635	3.20E-10	6.07807	7.59242	6.58E-10	4.51366
11	Monostearin	0.22807	4.95E-09	2.833215	2.07154	0.00015	2.29247	4.3846	4.95E-09	2.59887
12	Onapristone	0.38163	0.00130	1.65142	7.81206	3.15E-09	5.08336	10.66641	1.29E-10	4.48954
13	Oxamide	0.11328	0.00415	2.49446	8.82767	0.00415	2.82369	8.8278	0.00415	2.29857
14	Oxibendazole	0.02766	2.06E-16	6.29175	0.04339	0.00041	3.81391	0.02537	1.99E-05	3.30990
15	Paliperidone	0.39303	0.03442	1.24023	4.20549	0.00045	2.41296	3.35587	0.00211	2.05907
16	Sulfolithocholic acid	0.28332	7.03E-05	1.75722	2.55579	0.00303	1.58137	2.83680	0.00071	1.16347
17	Sunitinib	6.41533	0.04036	1.92767	2.75971	0.00555	2.27649	3.13116	0.00191	1.66104
18	Tributyl citrate acetate	4.02752	0.03744	1.67060	2.28837	0.00515	1.87899	2.08951	0.02348	1.08023

## Discussion

WHO's End TB Strategy calls for the early diagnosis of TB, highlighting the critical role of laboratories in the post-2015 era in rapidly and accurately detecting TB (21). However, there is a lack of a gold standard for diagnosing latent tuberculosis infection, nor is there a uniform laboratory specification for the discharge of TB patients (6). Consequently, the screening of new diagnostic biomarkers has the potential to improve diagnostic accuracy and may provide unified diagnostic criteria for latent tuberculosis infection and the discharge of TB patients.

It is worth noting that metabolites represent the effects of cell viability and external exposure; therefore, the wealth of small-molecule metabolite data represented by individual metabolomes can generate key pathological insights. Metabolomics has also been widely used in TB over the past few years (12, 22–24). In 2014 and 2015, Mrinal et al. (25) and Sebabrata et al. (26) reported the application of liquid chromatography–mass spectrometry and gas chromatography mass spectrometry methods to identify the metabolites in urine samples of TB patients, respectively. Subsequently, many studies based on LC-MS approaches reported small molecule metabolites can be used as biomarkers for pulmonary TB in plasma, such as L-Histidine, arachidonic acid, biliverdin, L-cysteine-glutathione disulfide, Xanthine, 4-Pyridoxate, and D-glutamic acid (3). However, these studies only used LC-MS to explore the role of differential metabolites in plasma in PTB, it is not yet possible to detect all compounds with LC-MS.

To identify more metabolites, we screened differential metabolites in the serum of untreated PTB patients, 2-month-treated PTB patients, cured TB patients, latent tuberculosis infection, and healthy controls by GC-MS and LC-MS/MS. The GC-MS method has increased sensitivity to detect very small volatile or semi-volatile compounds in biological samples. To our knowledge, there have been few previous untargeted metabolomics studies of serum samples from TB patients that combined GC-MS and LC-MS/MS, especially for evaluating the efficacy of anti-TB treatment. Seven previously unreported metabolites were observed in latent tuberculosis infection, including 3-oxopalmitic acid, akeboside Ste, sulfolithocholic acid, 2-Decylfuran, (4,8,8-trimethyldecahydro-1,4-methanoazulen-9-yl)methanol, D-(+)-Camphor, and 2-methylaminoadenosine. In this study, seven metabolites were increased in the serum of latent tuberculosis infection and untreated TB patients. It has been reported that in the inflammatory response, the palmitic acid and its derivatives in the endoplasmic reticulum (ER), on the one hand, can increase the reactive oxygen species (ROS) generation, leading to cell death; on the other hand, they also drive the activation of NF-κB and NLRP3, facilitating the release of proinflammatory cytokine by monocytes/macrophages (27, 28). José Marcos Sanches et al. had also shown that certain potential lipid biomarkers in macrophages, such as palmitic acid and PE (16:0/0:0), are released after NLRP3 activation that can modulate the inflammatory responses in the damaged tissue (29). 3-oxopalmitic acid is oxo-fatty acid comprising palmitic acid (PA) having an oxo group at the 3-position, an intermediate in fatty acid biosynthesis, and has functional parent palmitic acid. Our study also showed that 3-oxopalmitic acid was upregulated after Mtb infection, further indicating that Mtb may depend on fatty acid metabolism to maintain chronic infection.

Two secondary metabolites (2-methylaminoadenosine and 2-decylfuran) showed higher expression in latent tuberculosis infection and untreated TB patients compared with the healthy group. 2-methylaminoadenosine is a purine nucleoside. Currently, the natural products and derivatives of purine nucleosides have been developed as drugs for their unique biochemical properties and capabilities (30). 2-decylfuran is a member of the class of furans that is furan in which the hydrogen at position 2 is replaced by a decyl group. Furan exerts its antibacterial activity through selective inhibition of microbial growth and modification of enzymes (31). Our study showed that the 2-methylaminoadenosine and 2-decylfuran content increased rapidly after Mtb infection, but recovered after 2/6 months of anti-tuberculosis treatment. In most cases, secondary metabolites are metabolically or physiologically non-essential metabolites that may serve a role as defense or signaling molecules. This indicated that 2-methylaminoadenosine and 2-decylfuran are closely related to the survival of Mtb; therefore, we suspect that these two metabolites reflect the concentration of Mtb, *in vivo* after the intensive treatment phase. Camphor is a cyclic monoterpene ketone, which has been widely used as a chiral, enantiopure starting material in natural product synthesis (32). The study reported that a series of new amidoalcohols and amido diols were designed on the base of the camphor scaffold and evaluated for their *in vitro* activity against Mtb strains, and they showed 25 times higher activity than the classical anti-TB drug ethambutol (33). Our study showed that the D-(+)-camphor content increased rapidly after being infected with Mtb, indicating that camphor as a carrier has a strong bactericidal effect. Akeboside Ste is a triterpenoid, the studies reported that triterpenoids have various pharmacological activities, including anti-inflammatory, anti-allergic, anti-microbial, anti-angiogenic, etc. (34). Mtb stimulates the body's metabolism to produce akeboside Ste. As an anti-bacterial active substance, akeboside Ste would fight against tuberculosis quickly once Mtb is released from the body. Sulfolithocholic acid is the sulfated product of lithocholic acid, a secondary bile acid produced by microbiota (35). Recent reports suggest that sulfolithocholic acid is a potential marker for pancreatic fat (36). Our study showed that the level of akeboside Ste and sulfolithocholic acid significantly increased in latent tuberculosis infection and untreated TB patients, but both returned to a normal level after 2/6 months of TB treatment. This indicated that akeboside Ste and sulfolithocholic acid may be associated with the severity of Mtb infection and could reflect the change in the body's immunity to Mtb. Based on a literature review very few articles have been published on (4,8,8-trimethyldecahydro-1,4-methanoazulen-9-yl)methanol. Studies showed that through physiological and metabolic clearance mechanisms, methanol remains at a low physiological level in healthy people, but increased levels of methanol were detected in the blood of patients with nervous systems disorders and the elderly (37). Our study showed that (4,8,8-trimethyldecahydro-1,4-methanoazulen-9-yl)methanol was upregulated after Mtb infection and recovered after curing TB, suggesting a disruption of the genetic and biochemical mechanisms that are responsible for maintaining low methanol levels.

In conclusion, comparative serum metabolome analysis using GC-MS and LC-MS/MS demonstrated that differences do exist among untreated TB patients, two-month treated PTB patients, cured TB, latent tuberculosis infection, and healthy subjects. 3-oxopalmitic acid, akeboside Ste, sulfolithocholic acid, 2-decylfuran, (4,8,8-trimethyldecahydro-1,4-methanoazulen-9-yl)methanol, D-(+)-camphor, and 2-methylaminoadenosine may serve as potential biomarkers for latent tuberculosis infection and cured TB patients. These metabolites may reflect the severity of MTB infection in patients with TB and the strength of the body's immune defense. New metabolites from latent tuberculosis infection presented in this study, to our knowledge, have not been reported before. Moreover, these metabolites may also provide a promising approach to predict a good therapeutic outcome. Whether these metabolites add value to the prediction of latent tuberculosis infection and cured TB patients will require further study and validation in separate cohorts. Our study provided more detailed experimental data for developing laboratory standards for evaluating latent tuberculosis infection and cured PTB.

## Data availability statement

The datasets presented in this study can be found in online repositories. The names of the repository/repositories and accession number(s) can be found in the article/Supplementary material. Further inquiries can be directed to the corresponding author/s.

## Ethics statement

The study was approved by the Ethics Committee of the Center for Tuberculosis Control of Guangdong Province, China. Written informed consent was obtained from all subjects prior to blood sample collection.

## Author contributions

YL, WWe, and QT conceived, designed, and supervised the overall study. WWe, XW, and QT acquired funding, supervised, and administered the project. XW and WWe coordinated the study. XW, YL, and WWa collected the samples and the clinical supervision. YZ, JW, and LX processed the samples and performed the experiments. CZ, MY, XC, JZ, and LC analyzed the data. ZW, WWe, and XW wrote the article. JZ revised the article. All authors read and approved the final manuscript.

## Funding

This work was supported by the Major Infectious Disease Prevention and Control of the National Science and Technique Major Project (2018ZX10715004-002), the Dengfeng Plan High-level Hospital Construction Opening Project of Foshan Fourth People's Hospital (FSSYKF-2020015, FSSYKF-2020008), Guangdong Provincial Natural Science Foundation (2020A1515010658 and 2018A030310677), the Medical Scientific Research Foundation of Guangdong Province (A2020611), Youth Innovative Talents Project in Colleges and Universities in Guangdong Province (2021KQNCX208), and Medical Scientific Research Projects of Foshan Health Bureau (20210358).

## Conflict of interest

The authors declare that the research was conducted in the absence of any commercial or financial relationships that could be construed as a potential conflict of interest.

## Publisher's note

All claims expressed in this article are solely those of the authors and do not necessarily represent those of their affiliated organizations, or those of the publisher, the editors and the reviewers. Any product that may be evaluated in this article, or claim that may be made by its manufacturer, is not guaranteed or endorsed by the publisher.

## Supplementary material

Supplementary Figure 1 Identification of serum metabolites between uninfected healthy control (Un) and the untreated PTB group (T0). The PLS-DA model for the Un/T0 group in GC-MS **(A)**, LC-MS/MS (+) **(E)** and LC-MS/MS (–) **(I)**. The permutation test results for the Un/T0 group in GC-MS **(B)**, LC-MS/MS (+) **(F)** and LC-MS/MS (–) **(J)**; Volcanic map of differential metabolites for the Un/T0 group in GC-MS **(C)**, LC-MS/MS (+) **(G)** and LC-MS/MS (–) **(K)**. The abscissa: the fold change of Un/T0 group (base 2 logarithm). The ordinate: the *P*-value of Un/T0 group (base 10 logarithm). Red: significantly upregulated metabolites. Green: significantly downregulated metabolites. Gray: non-significant differential metabolites. Pathway analysis of differential metabolites for the Un/T0 group in GC-MS **(D)**, LC-MS/MS (+) **(H)**, and LC-MS/MS (–) **(L)**.

Supplementary Figure 2 Identification of serum metabolites between the untreated PTB group (T0) and the 2-month treated PTB group (T2). The PLS-DA model for the Un/T0 group in GC-MS **(A)**, LC-MS/MS (+) **(E)**, and LC-MS/MS (–) **(I)**. The permutation test results for the T0/T2 group in GC-MS **(B)**, LC-MS/MS (+) **(F)**, and LC-MS/MS (–) **(J)**. Volcanic map of differential metabolites for the T0/T2 group in GC-MS **(C)**, LC-MS/MS (+) **(G)**, and LC-MS/MS (–) **(K)**. The abscissa: the fold change of the T0/T2 group (base 2 logarithm). The ordinate: the *P*-value of the T0/T2 group (base 10 logarithm). Red: significantly upregulated metabolites. Green: significantly downregulated metabolites. Gray: non-significant differential metabolites. Pathway analysis of differential metabolites for the T0/T2 group in GC-MS **(D)**, LC-MS/MS (+) **(H)**, and LC-MS/MS (–) **(L)**.

Supplementary Figure 3 Changes in relative quantitative values of seven differential metabolites 3-oxopalmitic acid, akeboside ste, sulfolithocholic acid, 2-decylfuran, (4,8,8-trimethyldecahydro-1,4-methanoazulen-9-yl)methanol, d-(+)-camphor, and 2-methylaminoadenosine in the serum from healthy controls (Un and LTBI), the untreated PTB (T0), 2-month treated PTB (T2) and 6-month treated PTB. The *q*-value was calculated using the Kruskal–Wallis test and corrected for multiple comparisons by controlling the False Discovery Rate. ^*^*q* < 0.05; ^**^*q* < 0.01; ^***^*q* < 0.001.

Supplementary Figure 4 The flowchart of the subjects' screening and enrolling in this study. To study the relationship between tuberculosis and serum metabolome, we recruited 147 subjects that include 52 uninfected (Un), 40 LTBI, and 55 active TB patients without anti-TB therapy (T0). In addition, to detect the effect of periodic HRZE treatment on the serum metabolome, we tried to track TB patients in the T0 group over the course of anti-TB treatment, and finally succeeded in obtaining serum samples from 17 patients with complete standard treatment nodes, viz. 2 months (T2) and 6 months (T6). Next, we screened 17 volunteers of similar age to these 17 patients in each of the Un and LTBI groups. Two serum samples from the Ungroup and one serum from the LTBI group were found to have hemolysis during metabolite extraction and were discarded. Finally, 82 serum samples were used for the metabolome experiment.

Supplementary Table 1 Characteristics of the individuals in this study.

Supplementary Table 2 Serum metabolites identified between the LT group and the T0 group. The Variable Importance in the Projection (VIP) value of the first principal component of the PLS-DA model is used, and the VIP value represents the contribution rate of metabolite differences in different groups; The ratio of the mean values of all biological replicates in the comparison group; combined with the *P*-value of *T*-test to find differentially expressed metabolites, set the threshold to VIP > 1.0, the difference fold FC ≥ 2 or FC ≤ 0.5, and *P*-value < 0.05.

Supplementary Table 3 Serum metabolites identified between the Un group and the T0 group. The Variable Importance in the Projection (VIP) value of the first principal component of the PLS-DA model is used, and the VIP value represents the contribution rate of metabolite differences in different groups; The ratio of the mean values of all biological replicates in the comparison group; combined with the *P*-value of *T*-test to find differentially expressed metabolites, set the threshold to VIP > 1.0, the difference fold FC ≥ 2 or FC ≤ 0.5, and *P*-value < 0.05.

Supplementary Table 4 Serum metabolites identified between the T0 group and the T6 group (FC > 2.0, VIP > 1.0 and *P* < 0.05).

Supplementary Table 5 Serum metabolites identified between the T0 group and the T2 group (FC > 2.0, VIP > 1.0 and *P* < 0.05).

Supplementary Table 6 The total list of differential serum metabolites of T0 patients identified by LC-MS/MS and GC-MS, compared to LT and Un individuals (FC > 2.0, VIP > 1.0 and *P* < 0.05).

Supplementary Table 7 The relative amount of seven potential biomarkers shown in Figure 3 and Supplementary Figure 3.

Supplementary Table 8 KEGG pathway enriched by differential metabolites.

Supplementary Table 9 The function and classification of identified metabolites in this study were annotated by the HMDB database.

Supplementary Table 10 The function and classification of identified metabolites in this study were annotated by the KEGG database.

Supplementary Table 11 The function and classification of identified metabolites in this study were annotated by the LIPID MAPS database.
