# Supplementary figures and images for: Untargeted metabolomics of pulmonary tuberculosis patient serum reveals potential prognostic markers of both latent infection and outcome
Source: Front Public Health. 2022 Nov 15;10:962510. doi: 10.3389/fpubh.2022.962510 (PMC9705731; doi:10.3389/fpubh.2022.962510)

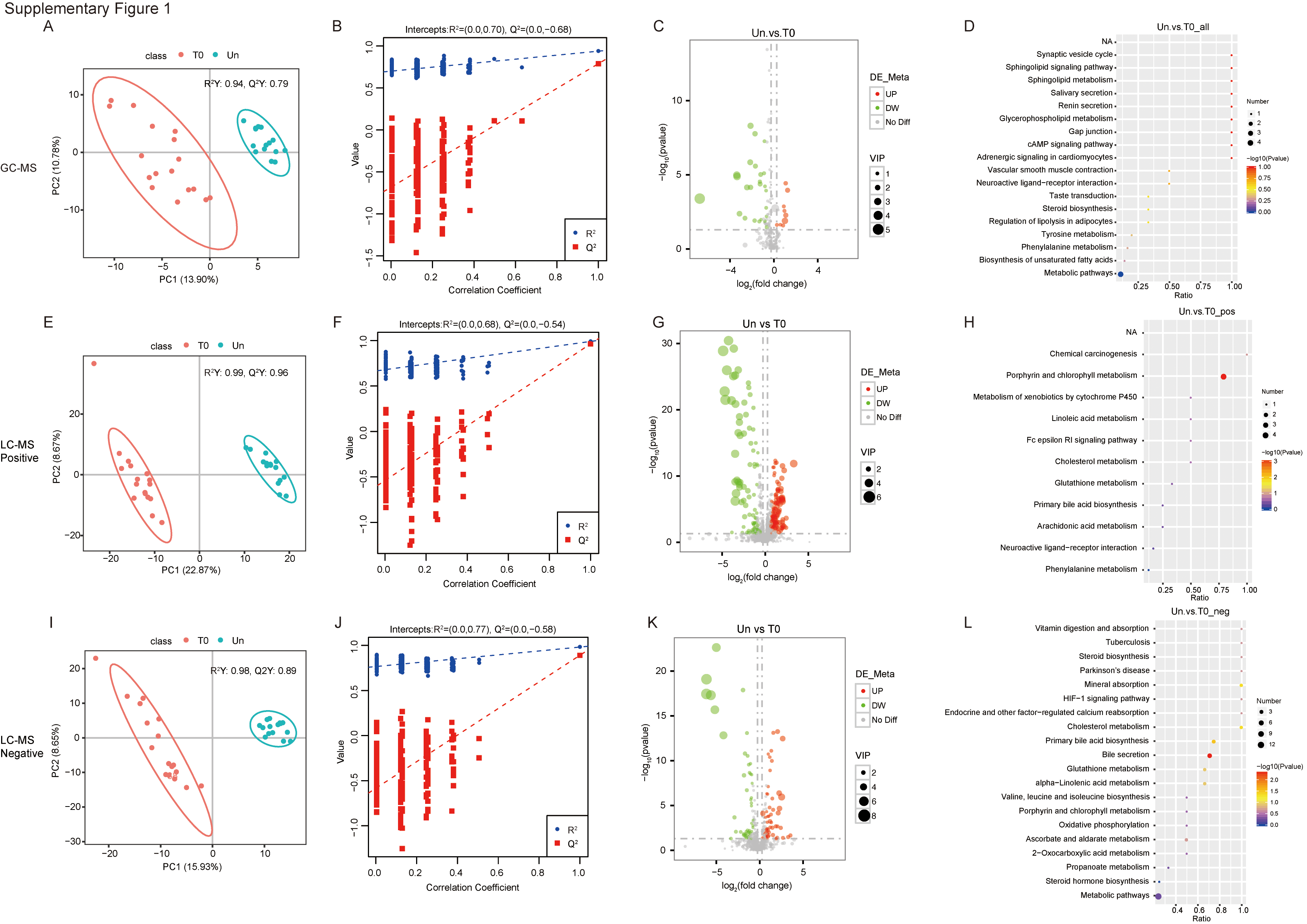

Supplement: Supplementary Figure 1 — Identification of serum metabolites between uninfected healthy control (Un) and the untreated PTB group (T0). The PLS-DA model for the Un/T0 group in GC-MS (A), LC-MS/MS (+) (E) and LC-MS/MS (–) (I). The permutation test results for the Un/T0 group in GC-MS (B), LC-MS/MS (+) (F) and LC-MS/MS (–) (J); Volcanic map of differential metabolites for the Un/T0 group in GC-MS (C), LC-MS/MS (+) (G) and LC-MS/MS (–) (K). The abscissa: the fold change of Un/T0 group (base 2 logarithm). The ordinate: the P-value of Un/T0 group (base 10 logarithm). Red: significantly upregulated metabolites. Green: significantly downregulated metabolites. Gray: non-significant differential metabolites. Pathway analysis of differential metabolites for the Un/T0 group in GC-MS (D), LC-MS/MS (+) (H), and LC-MS/MS (–) (L). [file Image_1.tif]

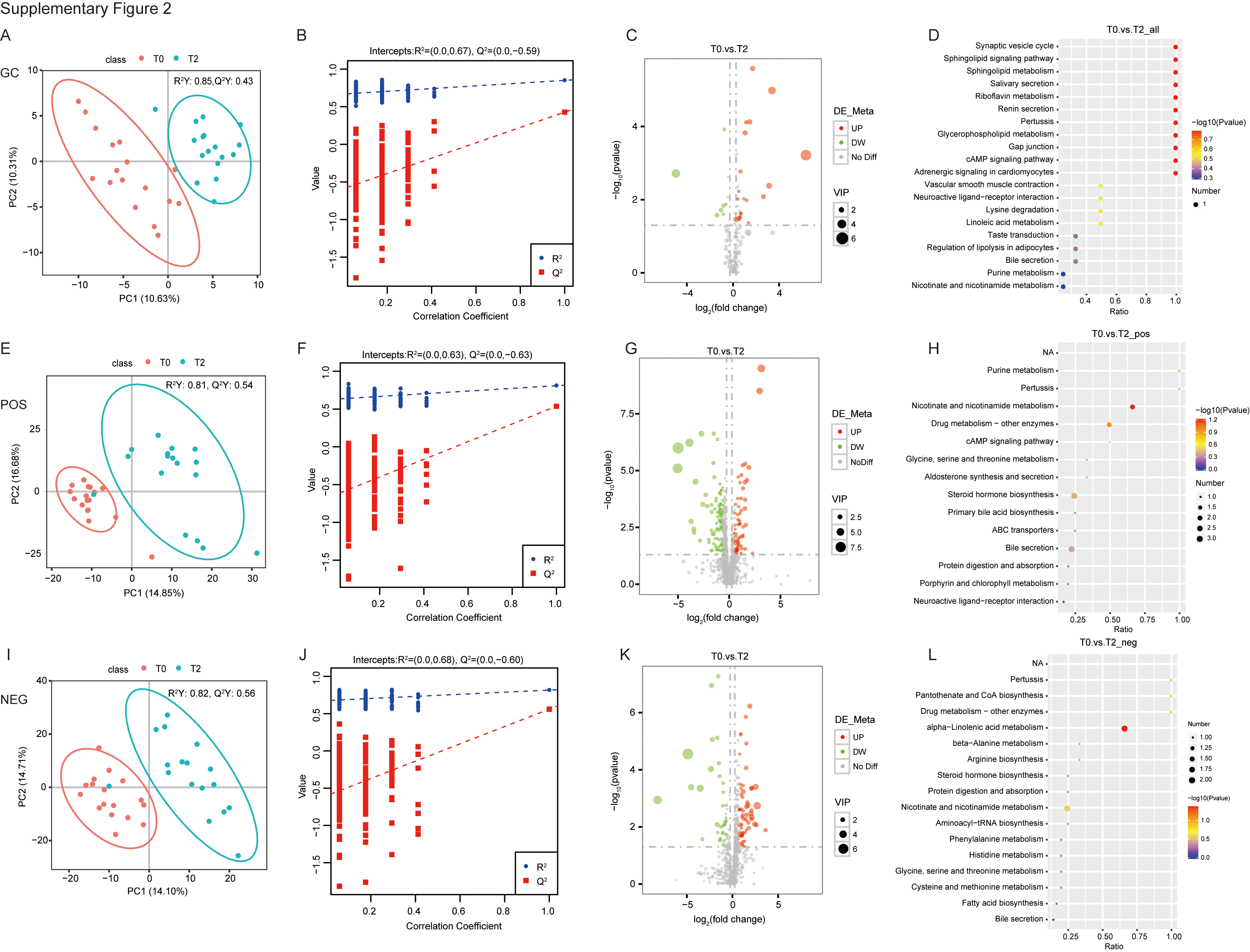

Supplement: Supplementary Figure 2 — Identification of serum metabolites between the untreated PTB group (T0) and the 2-month treated PTB group (T2). The PLS-DA model for the Un/T0 group in GC-MS (A), LC-MS/MS (+) (E), and LC-MS/MS (–) (I). The permutation test results for the T0/T2 group in GC-MS (B), LC-MS/MS (+) (F), and LC-MS/MS (–) (J). Volcanic map of differential metabolites for the T0/T2 group in GC-MS (C), LC-MS/MS (+) (G), and LC-MS/MS (–) (K). The abscissa: the fold change of the T0/T2 group (base 2 logarithm). The ordinate: the P-value of the T0/T2 group (base 10 logarithm). Red: significantly upregulated metabolites. Green: significantly downregulated metabolites. Gray: non-significant differential metabolites. Pathway analysis of differential metabolites for the T0/T2 group in GC-MS (D), LC-MS/MS (+) (H), and LC-MS/MS (–) (L). [file Image_2.tif]

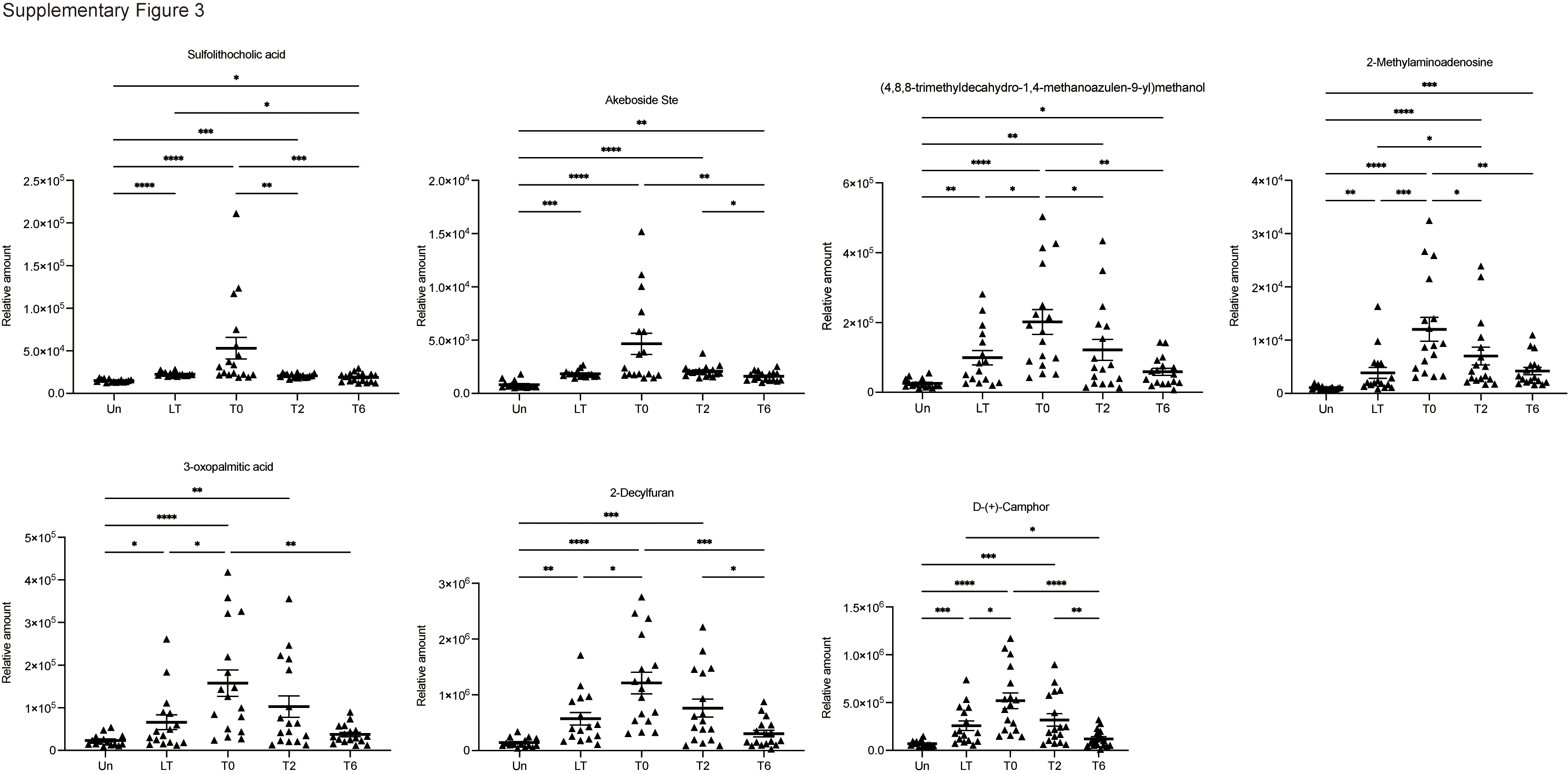

Supplement: Supplementary Figure 3 — Changes in relative quantitative values of seven differential metabolites 3-oxopalmitic acid, akeboside ste, sulfolithocholic acid, 2-decylfuran, (4,8,8-trimethyldecahydro-1,4-methanoazulen-9-yl)methanol, d-(+)-camphor, and 2-methylaminoadenosine in the serum from healthy controls (Un and LTBI), the untreated PTB (T0), 2-month treated PTB (T2) and 6-month treated PTB. The q-value was calculated using the Kruskal–Wallis test and corrected for multiple comparisons by controlling the False Discovery Rate. *q < 0.05; **q < 0.01; ***q < 0.001. [file Image_3.tif]

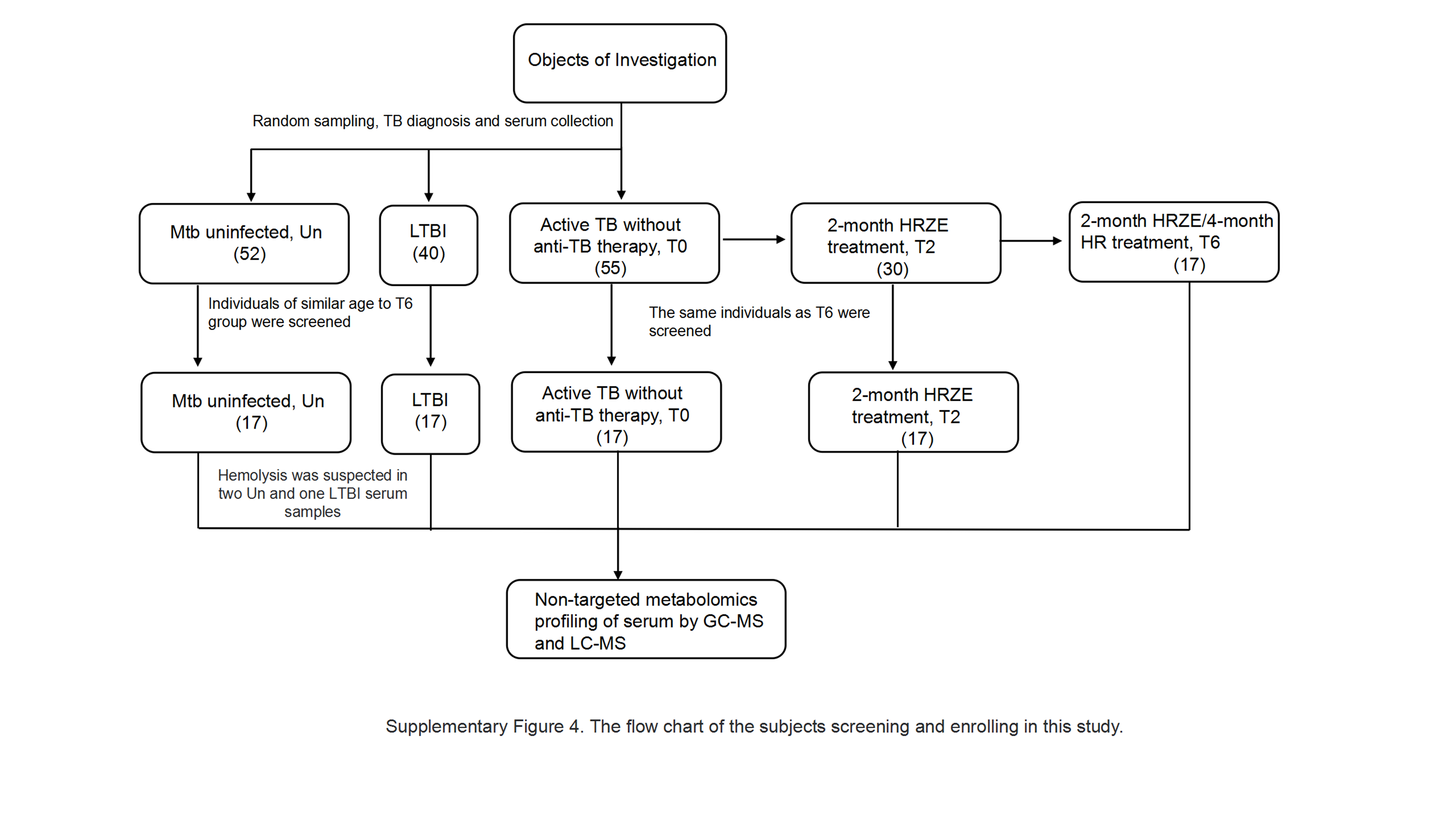

Supplement: Supplementary Figure 4 — The flowchart of the subjects' screening and enrolling in this study. To study the relationship between tuberculosis and serum metabolome, we recruited 147 subjects that include 52 uninfected (Un), 40 LTBI, and 55 active TB patients without anti-TB therapy (T0). In addition, to detect the effect of periodic HRZE treatment on the serum metabolome, we tried to track TB patients in the T0 group over the course of anti-TB treatment, and finally succeeded in obtaining serum samples from 17 patients with complete standard treatment nodes, viz. 2 months (T2) and 6 months (T6). Next, we screened 17 volunteers of similar age to these 17 patients in each of the Un and LTBI groups. Two serum samples from the Ungroup and one serum from the LTBI group were found to have hemolysis during metabolite extraction and were discarded. Finally, 82 serum samples were used for the metabolome experiment. [file Image_4.tif]
